# Shining Light on the Unusual: A Case Report of a Rare Presentation of Abdominal Wall Erysipelas

**DOI:** 10.7759/cureus.62628

**Published:** 2024-06-18

**Authors:** Wassim Abouzeid, Nibras Yar Khan, Aharnish Patel, Nyan A Bethel, Charity Iheagwara, Jihad Slim

**Affiliations:** 1 Internal Medicine, Saint Michael's Medical Center, Newark, USA; 2 Internal Medicine, New York Medical College, Saint Michael's Medical Center, Newark, USA; 3 Infectious Diseases, Saint Michael's Medical Center, Newark, USA

**Keywords:** streptococcus group a infection, streptococcus pyogenes, skin infections, erysipelas, abdominal wall erysipelas

## Abstract

We present a clinical case detailing the presentation of erysipelas in a 52-year-old immunocompetent female, wherein the infection displayed an unusual localization encompassing the skin of the anterior abdominal area and breast. The patient exhibited a favorable response to medical treatment. It is paramount to underscore the significance of recognizing such cases, which demand a heightened level of clinical suspicion to facilitate swift diagnosis and effective management strategies.

## Introduction

Erysipelas is a skin infection involving the dermis layer of the skin, but it may also extend to the superficial cutaneous lymphatics. It is characterized by an area of erythema that is well-demarcated and raised with sharp borders, unlike cellulitis. Despite its characteristic appearance, erysipelas frequently masquerades as other dermatological conditions, leading to diagnostic delays and inappropriate treatment approaches [1]. Erysipelas can affect all age groups but is most common in the extremes of age. A few studies have shown a predilection for females. This skin infection is caused by *Streptococcus* group A infection and most commonly occurs on the lower limbs. The second most common site is the face. It usually does not develop on the abdomen unless it is a surgical site infection or through a breach in the skin via trauma or insect bite. Erysipelas on the abdominal wall is very rare. We present a unique case of erysipelas on the abdominal wall and involving the patient’s breast [2].

## Case presentation

A 52-year-old female presented to the hospital complaining of subjective fever, chills, abdominal wall erythema, and pain for a one-day duration, with a past medical history of hypertension and diverticulosis and a past surgical history of bilateral breast reduction that was done two years ago. A day prior to admission, the patient mentioned that she started feeling fatigued along with anorexia and body aches with an area of mild erythema of the skin surrounding the umbilicus, for which she took acetaminophen, which resulted in mild relief. However, on the next day, she noticed severe diffuse erythema involving her entire abdominal wall and bilateral inframammary areas along with pain but denied any itching or prior history of skin infection.

One year ago, the patient had an umbilical piercing for a belly ring, and she mentioned that this was the first time she noticed any signs of inflammation in this area since the piercing. 

**Figure 1:**
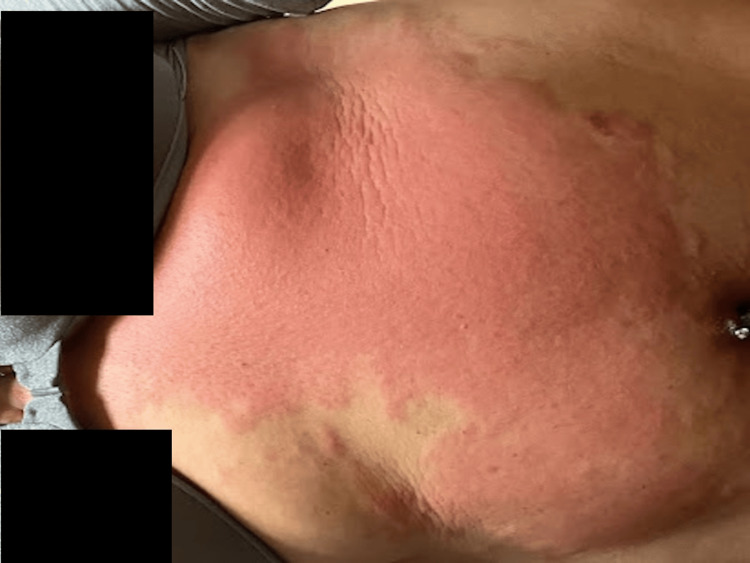
On the day of presentation, the patient showed diffuse abdominal wall erythema involving the anterior abdominal wall and bilateral inframammary folds

On admission, the patient had a low-grade fever of 99.1°F with normal rest of vital signs; blood pressure was 125/84 mmHg, heart rate was 72 beats per minute, and respiratory rate was 16 breaths per minute. Physical examination showed diffuse erythema and tenderness involving the anterior abdominal wall with no papules, vesicles, or pustules, as shown in Figure 1. Initial significant laboratory results are shown in Table 1.

**Table 1:** Significant laboratory results

	Value	Reference range
WBCs	14.6 × 10^3^/uL	4.4-11 × 10^3^/uL
Neutrophils	11.2 × 10^3^/uL	1.7-7 × 10^3^/uL
Lactic acid	1.05 mmol/L	0-2 mmol/L
CRP	16.1 mg/dL	0-0.8 mg/dL
HIV Ag/Ab	Negative	Negative
Blood culture	No growth after five days	No growth
Anti-DNAse B strep	240 U/mL	0-120 U/mL

**Figure 2:**
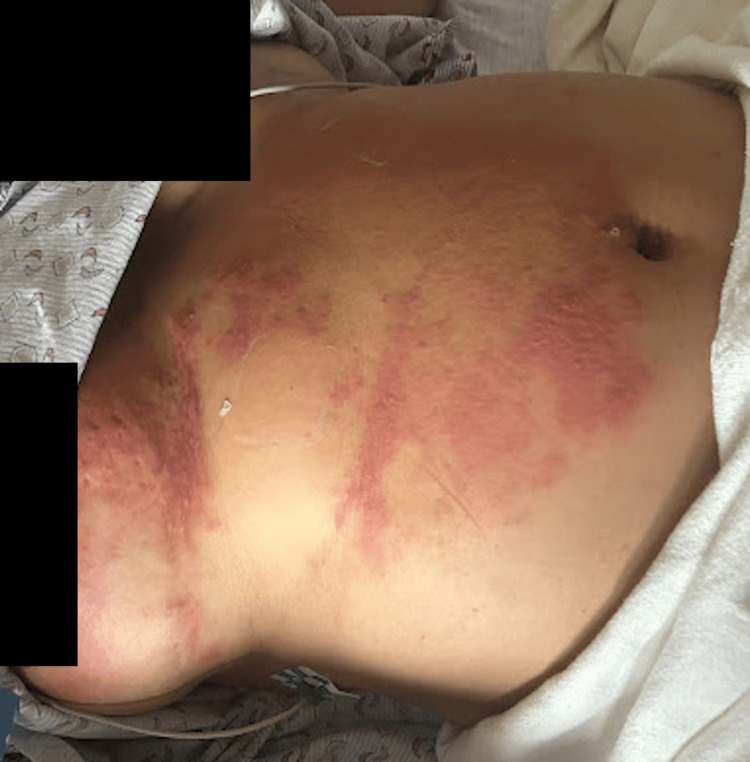
On day 2, the patient showed improvement in previously shown erythema

A CT abdomen with IV contrast was done and showed focal areas of fat stranding involving the anterior abdominal wall and left the para umbilical region. Diagnosis of severe abdominal wall erysipelas was made, the belly ring was removed, and the patient was started on intravenous cefazolin 1 g every eight hours. Over the next two days, the patient showed clinical improvement of erythema and pain, as shown in Figure 2, as well as normalization of inflammatory markers except for CRP, which was downtrend to 2.5 mg/dL.

The patient was later discharged on a five-day course of amoxicillin-clavulanic acid, and two weeks later, on follow-up, a complete resolution of the erythematous rash was noted.

## Discussion

*Streptococcus pyogenes* causes most of the facial infections, while erysipelas on the legs could also be caused by non-group A streptococci. These bacteria mainly enter the skin through a port of entry like a surgical site, insect bite site, eczema, or athlete’s foot and affect the superficial epidermis. Risk factors like pre-existing lymphedema, venous stasis, immunocompromised states like uncontrolled diabetes, liver disease, intravenous drug use, and HIV could predispose to erysipelas [2]. 

Erysipelas is an acute bacterial superficial skin infection involving the dermis and the hypodermis that characteristically extends into the superficial cutaneous lymphatics. It presents as a tender, intensely erythematous, indurated plaque with a sharply demarcated border, which helps differentiate it from other skin infections [3]. 

The overall incidence of erysipelas has significantly decreased over the past few decades due to improved sanitation and antibiotic use. However, some studies have suggested a greater incidence in females and those of extreme age. Historically, erysipelas occurred on the face, but currently, most cases involve the legs, and it is relatively less common in other sites like the abdominal wall [3].

Diagnosis of erysipelas is primarily clinical, with characteristic sudden onset of erythema, edema, pain, and warmth in the affected area, often associated with fever, chills, and malaise. No specific tests or imaging is needed to diagnose erysipelas; however, a few lab parameters like white blood cell counts, sedimentation rate, and CRP may be helpful in predicting prognosis or evolving complications [4,5]. 

Most patients with erysipelas can be treated as an outpatient with oral antibiotics like penicillin, cephalexin, or clindamycin. If systemic signs of infection are present, it may be treated with IV cefazolin, clindamycin, or Unasyn, usually for five days, but may be extended to 10 days in moderate to severe cases [6].

Systemic complications are rare when not treated promptly, but sepsis is reported in a minor percentage [7,8]. Local complications like abscess formation, necrosis, bubbles, and hemorrhagic purpura have been noted in about 30% of patients hospitalized for erysipelas and are mostly associated with a more severe condition [9,10]

Upon reviewing the existing literature, we found no previously reported cases of abdominal erysipelas. In this case, the patient’s history of recent abdominal wall erythema and subjective fever, chills, and tenderness prompted medical attention. We think that the belly button piercing was the port of entry for group A streptococcus in our patient. The recent onset of symptoms following fatigue, anorexia, and body aches suggested a systemic inflammatory response, likely triggered by the localized infection that improved rapidly with antibiotic therapy and supportive care. It is noteworthy that prompt recognition and initiation of appropriate antibiotics are crucial to prevent complications associated with this condition.

## Conclusions

Erysipelas is a common skin infection that usually occurs in the lower extremities and face. It can present at unusual sites, especially if there is a breach in the skin barrier, creating a portal of entry for the microbes. Our case shows that it can occur at such an unusual site as the abdomen, and this underscores the importance of a thorough physical examination, specifically looking for body piercing that could be the port of entry for *S. pyogenes*, as well as meticulous history-taking and high clinical suspicion that will prompt swift diagnosis and management.
